# Endoscopic anterior fundoplication with the Medigus Ultrasonic Surgical Endostapler (MUSE™) for gastroesophageal reflux disease: 6-month results from a multi-center prospective trial

**DOI:** 10.1007/s00464-014-3731-3

**Published:** 2014-08-19

**Authors:** Johannes Zacherl, Aviel Roy-Shapira, Luigi Bonavina, Amol Bapaye, Ralf Kiesslich, Sebastian F. Schoppmann, William R. Kessler, Don J. Selzer, Ryan C. Broderick, Glen A. Lehman, Santiago Horgan

**Affiliations:** 1 Department of General Surgery, Herz Jesu Krankenhaus, Vienna, Austria; 2 Department of Surgery A, Soroka University Hospital, Beer Sheva, Israel; 3 Department of Surgery IRCCS Policlinico San Donato, University of Milan School of Medicine Director, Milan, Italy; 4 Department of Digestive Diseases & Endoscopy, Deenanath Mangeshkar Hospital & Research Center, Pune, India; 5 Department of Internal Medicine and Gastroenterology, St. Marienkrankenhaus Frankfurt, Frankfurt, Germany; 6 Department of Surgery Comprehensive Cancer Center Vienna GET-Unit, Medical University of Vienna, Vienna, Austria; 7 Division of Gastroenterology/Hepatology, Department of Medicine, Indiana University School of Medicine, Indianapolis, IN USA; 8 Division of General Surgery, Department of Surgery, Indiana University School of Medicine, Indianapolis, IN USA; 9 Department of Surgery, Division of Minimally Invasive Surgery, Center for the Future of Surgery, University of California San Diego, San Diego, CA USA

**Keywords:** Gastroesophageal reflux disease, Anterior fundoplication, Proton pump inhibitors, Nissen fundoplication

## Abstract

**Background:**

Both long-term proton pump inhibitor (PPI) use and surgical fundoplication have potential drawbacks as treatments for chronic gastroesophageal reflux disease (GERD). This multi-center, prospective study evaluated the clinical experiences of 69 patients who received an alternative treatment: endoscopic anterior fundoplication with a video- and ultrasound-guided transoral surgical stapler.

**Methods:**

Patients with well-categorized GERD were enrolled at six international sites. Efficacy data was compared at baseline and at 6 months post-procedure. The primary endpoint was a ≥50 % improvement in GERD health-related quality of life (HRQL) score. Secondary endpoints were elimination or ≥50 % reduction in dose of PPI medication and reduction of total acid exposure on esophageal pH probe monitoring. A safety evaluation was performed at time 0 and weeks 1, 4, 12, and 6 months.

**Results:**

66 patients completed follow-up. Six months after the procedure, the GERD-HRQL score improved by >50 % off PPI in 73 % (48/66) of patients (95 % CI 60–83 %). Forty-two patients (64.6 %) were no longer using daily PPI medication. Of the 23 patients who continued to take PPI following the procedure, 13 (56.5 %) reported a ≥50 % reduction in dose. The mean percent of total time with esophageal pH <4.0 decreased from baseline to 6 months (P < 0.001). Common adverse events were peri-operative chest discomfort and sore throat. Two severe adverse events requiring intervention occurred in the first 24 subjects, no further esophageal injury or leaks were reported in the remaining 48 enrolled subjects.

**Conclusions:**

The initial 6-month data reported in this study demonstrate safety and efficacy of this endoscopic plication device. Early experience with the device necessitated procedure and device changes to improve safety, with improved results in the later portion of the study. Continued assessment of durability and safety are ongoing in a three-year follow-up study of this patient group.

Gastroesophageal reflux disease (GERD) is typically the result of lower esophageal sphincter (LES) dysfunction caused by inappropriate transient LES relaxation or diminution of resting basal pressure [1]. GERD is a chronic, relapsing disease with often under-appreciated adverse impacts on daily living, work productivity and health-related quality of life [2]. Consequently, a long-term management plan is necessary for most patients.

The daily use of proton pump inhibitors (PPI) is generally effective, although 20–30 % of PPI users are not entirely satisfied with this treatment [3, 4] and up to 40 % of patients do not respond or have an incomplete response to PPI therapy [5–8]. While PPI therapy can heal esophagitis and manage acid-related heartburn symptoms, it is less effective for extraesophageal symptoms of GERD and in patients with symptomatic regurgitation [9–13]. Patients may require increased PPI dosage and other ancillary medications to control their symptoms [8, 11]. Concerns about the potential side effects of chronic PPI therapy include increased risk of bone fracture, infectious complications and interference with anti-platelet medications, and the absorption of vitamins and minerals (e.g., B12, calcium, magnesium, iron) [14–16].

The primary alternative to chronic PPI use is surgical fundoplication and most commonly laparoscopic Nissen fundoplication (LNF). When performed by experienced surgeons, long-term results are excellent [17]. Not infrequently, however, LNF patients experience problematic side effects of gaseous bloating, flatulence, diarrhea, dysphagia, and the inability to belch or vomit [18, 19]. Incisional hernias have been reported in up to 3 % of procedures performed at centers of excellence [20]. There is a broad desire to develop less invasive techniques that address the GERD symptoms, but reduce the likelihood of dysphagia and bloating symptoms and do not require abdominal incision.

Endoscopic anterior fundoplication using a novel transoral endoscopic device (MUSE™, formerly called SRS, Medigus, Omer, Israel) has been evaluated as an alternative GERD therapy. Shown to be safe and technically facile in animal studies [21], the fundoplication is created transorally using a video- and ultrasound-guided surgical stapler. This report describes a multicenter, prospective clinical study to assess the 6-month safety and efficacy of this procedure which was used to treat 69 patients with GERD.

## Materials and methods

### Patients

The research protocol and endpoints were designed in close cooperation with US Food and Drug Administration (FDA) as an Investigational Device Exemption (IDE) study, approved by the institutional review board at each study center and registered at ClinicalTrials.gov (Identifier: NCT00734747). An independent Data Safety Monitoring Board (DSMB) advised the sponsor regarding safety of trial patients as well as the continuing validity and scientific merit of the trial. Training for each investigator consisted of bench top and live porcine procedures, and all procedures were attended by sponsor medical and engineering personnel. There were no additional training procedures prior to study enrollment at any site. Six international centers (3 in Europe, 1 in India, and 2 in the US) participated. Written informed consent was obtained from all participants. Patients were recruited directly from the investigator’s practice or in some cases approved advertisements. The study included patients aged 18–70 years with ≥2 years of documented GERD symptoms and ≥6 months of continuous PPI therapy. All subjects were candidates for LNF, according to guidelines of the Society of American Gastrointestinal and Endoscopic Surgeons (www.sages.org). Pathologic reflux (off PPI therapy) was confirmed by ambulatory esophageal pH monitoring during baseline evaluation. Patients must demonstrate significant GERD and PPI response by means of an abnormal GERD- health-related quality of life (HRQL) questionnaire score of ≥20 while off PPIs, and GERD-HRQL score improvement of at least 6 points while on PPIs. Patients were excluded if they had a body mass index >35, or substantial co-morbidities (e.g., heart disease, diabetes, cancer, previous gastric surgery). Endoscopic exclusion criteria included findings of hiatal hernia ≥3 cm, Barrett’s esophagus, Los Angeles grade IV esophagitis or esophageal luminal narrowing including stricture, ring, or web.

Each patient’s pre-procedure baseline data were compared to data collected 6 months post-procedure to evaluate procedure efficacy, defined as its effect on GERD-HRQL [22], intra-esophageal acid exposure, PPI use and anatomical change. Procedure safety was determined by evaluation of all treatment-related adverse events.

### Device and procedure

**Fig. 1:**
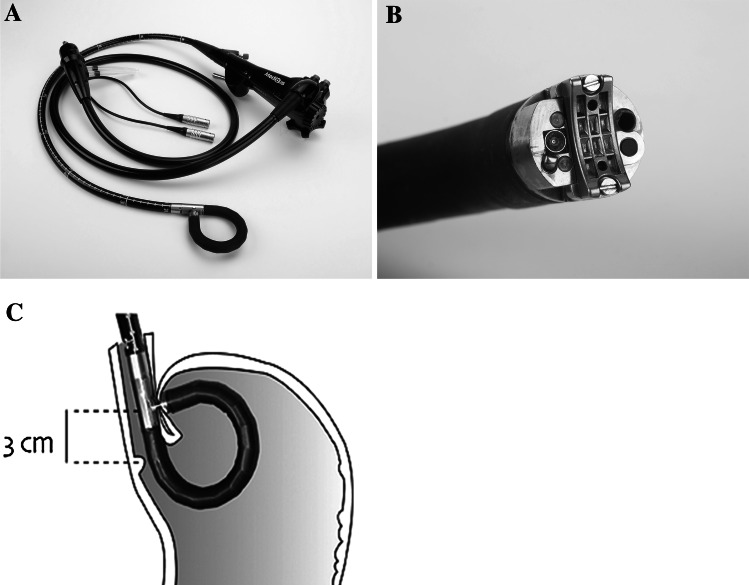
A–C Medigus transoral surgical stapler:** A** Full flexible endostapler, OD 15.5 mm** B** Distal tip** C** positioning of cartridge 3 cm proximal to gastroesophageal junction for stapling

The device consists of a light source, control unit, and flexible surgical endostapler which resembles an endoscope. The endostapler, designed to be operated by a single user, includes a handle with controls, a long (80 cm) flexible shaft, a short (5 cm) rigid section holding a cartridge with 5 standard 4.8 mm titanium surgical staples, a ratchet controlled one-way articulating section, and a distal tip (Fig. 1A). The distal tip (Fig. 1B) houses an anvil for bending the staples into a B shape, an ultrasonic transducer, a miniature video camera, a light source, and two fine (~21 gage) screws. The screws, secured by two nuts in the cartridge, provide a means for compressing tissue and a counterforce for bending the staples. The tip also contains suction/air insufflation and irrigation channels. The control unit interprets signals from the device and displays the resulting data on a video monitor, including the bending angle and force, ultrasound signal level, screw position, and the gap between the distal tip and the cartridge.

**Fig. 2:**
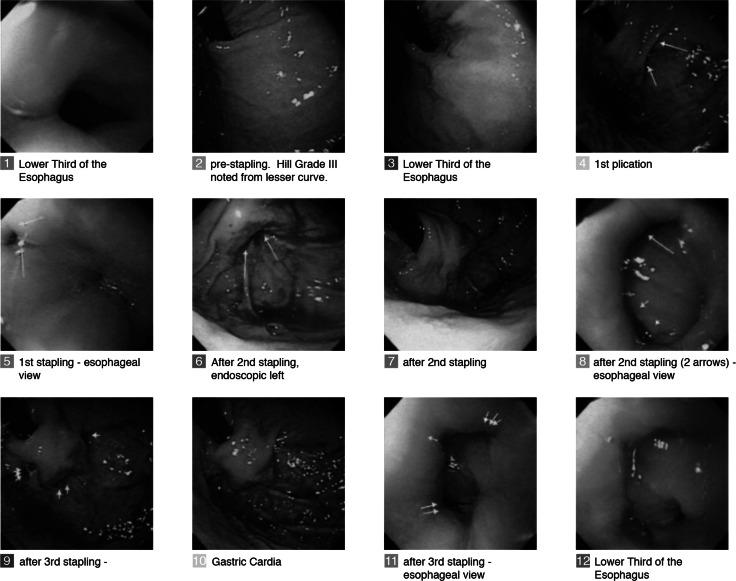
Step by step endoscopic images of a procedure involving the placement of three quintuplets of staples (15 staples total) to create an effective gastroesophageal flap valve

The procedures were performed under general anesthesia with endotracheal intubation in an operating room or a therapeutic endoscopy suite. Briefly, the transoral stapler was advanced into the stomach through an overtube (17 mm ID/19.5 mm OD) and retroflexed under direct video guidance. After identifying a stapling location, the stapler was gently pulled back to place the staple cartridge in the esophagus approximately 3 cm proximal to the gastroesophageal junction (Fig. 1C). The operator then used the articulation knob to bend the device tip to press the fundus against the esophagus. Next, the screws were deployed. As the tissues were compressed, and direct visualization was no longer possible, the ultrasonic range finder automatically engaged to display the tissue thickness. When the tissue thickness was 1.4–1.6 mm, the operator fired the stapler. Each firing delivers a quintuplet pattern of five standard 4.8 mm surgical staples simultaneously. The screws were retracted back into the tip of the device, and the stapler removed for reloading. The procedure was repeated to add additional quintuplets of staples, as allowed by the protocol. The goal was to mimic a partial anterior fundoplication, determined by a Hill Grade I valve [23] so that no esophageal mucosa is visible around the device in retrograde view (Fig. 2).

If a sliding hiatal hernia (SHH) was identified, ventilation with positive end expiratory pressure (PEEP) of 5 mmHg (6.8 cm H20) was applied, and gradually increased to 10 mmHg (13.6 cm H20) to reduce the stomach into the abdominal cavity. After the first 24 cases, the protocol was amended to reduce the pressure gradient between the abdominal and the thoracic cavity in order to prevent air leaks around the screws. Subsequently, all subjects were ventilated with a PEEP setting of 5 mmHg, after the orotracheal intubation. If SHH was still evident, PEEP was gradually increased to 10 mmHg until the hernia was reduced.

### Assessment of efficacy and safety

Each patient’s baseline data served as the comparison for post-procedure evaluation. A GERD-HRQL questionnaire was administered while patients were off PPI therapy for a minimum of 7 days. This validated instrument includes six heartburn-related items and questions relating to other GERD symptoms, medication use and satisfaction with present condition. The total GERD-HRQL score ranges from 0 to 50, with a higher score indicating more severe symptoms.

Secondary endpoints were elimination or ≥50 % reduction in dose of PPI medication, standardized to 40 mg/day of Omeprazole, reduction of total acid exposure on esophageal pH probe monitoring, and anatomical changes to the GEFV. Both PPI dose and frequency of PPI use were recorded at each study visit. At month 6, patients completed the GERD-HRQL questionnaire and underwent repeat esophageal pH measurement 5 cm above the manometric border of the LES, esophageal manometry and standard upper gastrointestinal endoscopy, after the patients were off PPI therapy for a minimum of 7 days. Esophageal pH measurements included total percent of time with pH <4, supine percent of time with pH < 4, number of episodes longer than 5 min, and the longest episode. Esophageal pH was considered normalized if the total time pH <4 was ≤4.2 % of time post-procedure [24]. LES pressure and length were recorded, as was peristaltic amplitude and residual LES pressure during relaxation. An evaluation of the Hill grade of the gastroesophageal flap valve (GEFV) as compared with baseline was performed. Satisfactory flap valve was defined as a grade I or II valve by the Hill classification.

Adverse events were evaluated at each visit at time 0, weeks 1, 4, 12, and month 6, as well as at any unscheduled visits. Serious adverse events were those that resulted in death, were life-threatening, or required prolongation of a current hospitalization. Per protocol, hospitalization was allowed for up to 72 h following the procedure. Hospitalization days beyond this period were recorded as a serious adverse event.

### Statistical analysis

The primary endpoint was a ≥50 % improvement in GERD-HRQL score in at least 53 % of subjects (lower bound of confidence interval). This success proportion was chosen to demonstrate a level of efficacy greater than that described by Cadiere et al. [25]. For proportions, exact binomial 95 % confidence intervals were constructed. Due to the nonparametric distribution of most of the continuous data, comparisons between baseline and post-procedure results were accomplished using Wilcoxon signed-rank test. The one exception was the evaluation of PPI dosage which was compared post-procedure to baseline using a paired *t* test. Differences in all tests were considered significant at the *P* < 0.05 level. The planned sample’s size was selected to provide an 80 % chance of a 95 % one-sided confidence interval excluding a success proportion of 0.53 if the actual proportion of success was equal to 0.68. This led to a minimum sample size of 63 patients; however, 72 were enrolled to allow for loss of follow-up or drop out.

## Results

### Baseline and procedural results

Seventy-two patients were consented and enrolled across 6 sites and served as the intent-to-treat (ITT) population for safety analyses (Table 1). A total of 8 patients were excluded from the 6-month efficacy analysis. Three patients were excluded upon esophageal screening and did not receive staple placement. Sixty-nine patients underwent the full procedure between May 2008 and November 2010. Three patients were treated, but upon review did not fulfill the inclusion/exclusion criteria related to initial GERD severity or improvement in score with PPI. Thus a total of 66 patients were included in the primary efficacy analysis. Two patients did not complete 6-month follow-up pH and manometry exams. Therefore, 64 subjects were included in the secondary efficacy analysis for those parameters (Table 2). The mean procedural time was 58 ± 38.6 min in the endoscopy units and 77.7 ± 42.4 min in the operating rooms. 36 procedures were performed by gastroenterologists and 33 by surgeons with established experience in advanced endoscopic procedures.

**Table 1:** Demographics and baseline characteristics of enrolled patients (*n* = 72)

	Female	Male
No. of patients	30	42
Age (year); median (range)	56.4 (27–71)	42.2 (24–74)
BMI (kg/m^2^); mean (range)	26.8 (18.9–35.6)	26.9 (19.3–34 2)

**Table 2:** Patient enrollment and disposition

	No.
Enrolled (ITT population for safety analysis)	72
Enrolled and did not complete procedure (no staples placed)	3
Excluded from primary efficacy analysis (did not meet inclusion criteria)	3
Total with baseline measures (per protocol and GERD-HRQI at 6 months)	66
Enrolled and refused ‘off-PPI’ testing at the 6-month follow-up visit	2
Total included in secondary efficacy analysis at 6 months	64

### Efficacy analysis

At 6 months, at least 50 % reduction in GERD-HRQL score (off PPI) from pre-procedure values was achieved in 48 of 66 patients (73, 95 % CI 60–83 %), thus the primary efficacy endpoint was met. Following the procedure, the median HRQL score (sum of questions 1–10) was significantly improved relative to the median baseline scores measured both on and off PPI medication (*P* < 0.001) (Table 3).

**Table 3:** Gastroesophageal disease health-related quality of life (GERD-HRQL) score and sub score analyses at baseline and 6 months post-procedure

	GERD-HRQL		
	Baseline (On PPi)	Baseline (Off PPI)	6 months (Off PPi)
*N*	66	66	64
Total score, mean (SD)	14.9 (7.5)	29.7 (6.2)	9.0 (9.1)
Total score, median	15	29*	6^†^
Heartburn sub score (Q1–6) mean (SD)	11.0 (5.8)	21.9 (3.6)	7.2 (7.3)
Heartburn sub score median	11	22*	5^†^

* Wilcoxon Signed-Rank test versus on PP1 baseline value, *P* < 0.001

^†^Wilcoxon Signed-Rank test versus off PP1 baseline value, *P* < 0.001

At baseline, nearly all (65/66) of procedure-treated patients were taking one or more PPI medications daily (one patient switched to high dose H2RA *after* screening). The average daily dose at baseline per patient was 58.5 ± 33.02 mg/day (standardized to 40 mg Omeprezole). At the 6-month follow-up, forty-two patients (64.6 %) were no longer using any daily PPI or other acid reducing medications. For the 23 patients, who continued to use PPI post-procedure and had 6-month follow-up values, a paired t test indicated a significant decrease in PPI usage of 31.3 mg/day (*t* = −3.88, df = 22, *P* = 0.001) (Table 4). Eighty-five percent (55/65) of the patients with daily PPI use at baseline reported a reduction in dose or frequency of at least 50 % post-procedure (Table 5). Use of occasional H2RA decreased in dose and frequency from 13 patients to 4 patients post-procedure.

**Table 4:** PPI utilization

	Baseline (*N* = 66)	6 month (*N* = 66)
Patients taking 1 or more PPI	65	23
Dose, mg/day; mean (SD)	58.5 (33.0)	31.3 (11.4)*
Dose, mg/day; mean	40	30

* P = 0.001 versus baseline (paired-1-test) only for the 23 patients that had baseline and follow-up values

**Table 5:** No. of patients using proton pump inhibitor (PPI) medication 6 months post-procedure

	*N* (%)	95 % CI^a^
PPI eliminated or reduced ≥50 %		
Off all PPIs	42/65 (65 %)	(52, 76)
Dose reduced <50 %	55/65 (85 %)	(74, 92)
Medication PPI not eliminated\not reduced ≥50 %		
Dose reduced <50 %	10/65 (15 %)	(8, 26)
Dose maintained	7/65 (11 %)	(4, 21)
Dose increased	2/65 (3 %)	(0, 11)

^a^Exact binomial 95 % CI

Of the 23 patients, who continued to take PPI medication following the procedure, 13 (56.5 %) reported at least a 50 % reduction in dose; seven patients did not change their PPI usage, two were taking higher doses after than before the procedure, and one reported PPI usage, but not the specific dosage.

### Additional analysis

#### GERD-HRQL sub score

**Fig. 3:**
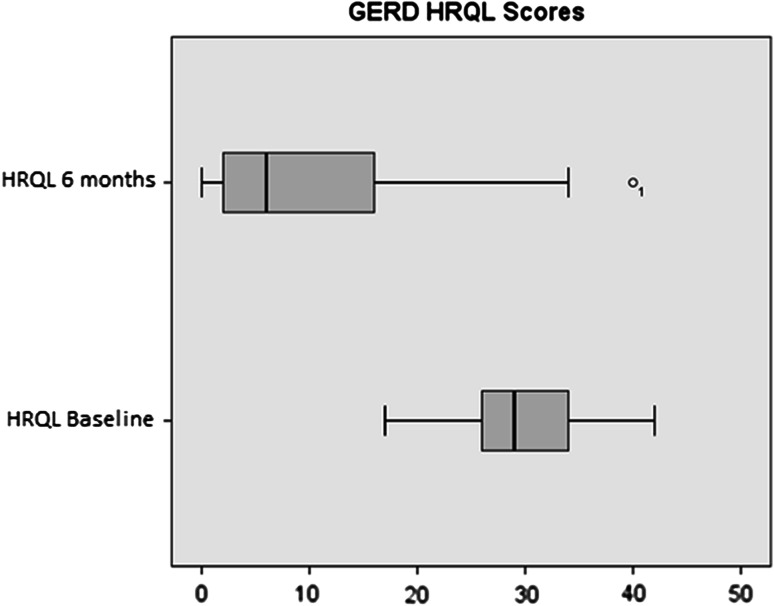
Boxplots of GERD-HRQL median scores at* baseline* (*N* = 66) and 6 months post-procedure (*N* = 64)

Exploratory analysis was performed on the quality of life scores in patients reported as the sum of questions specifically related to heartburn (Table 3). In an analysis of GERD-HQRL questions specific to heartburn (questions 1–6), median sub scores following the procedure were significantly improved when compared with baseline sub scores measured both on and off PPI medication (*P* < 0.001) (Fig. 3).

### Esophageal pH, manometry, and changes in GEFV (Hill Grade)

Esophageal pH (off PPI therapy) was measured in 66 patients pre-procedure and in 64 patients at 6 months post-procedure. Summary analyses indicated statistically significant reductions in the means for percent total time and upright time pH ≤4, as well as total number of episodes (Table 6). Analyses for each manometry endpoint are provided in Table 7. There were no significant changes in manometry parameters. In an analysis of GEFV Hill Grade scores, the proportions of patients with an unacceptable Hill Grade (>2) before (0.661, 43/65) and after (0.062, 4/65) the procedure were statistically different (*P* < 0.0001 per McNemar test) (Table 8).

**Table 6:** Summary of esophageal pH measures

Symptom	Baseline (off PPI)		6 months (off PPI)		*P* value*
	*n*	Mean (SD)	*n*	Mean (SD)	
pH ≤ 4 (%) total	66	10.9 (10.7)	64	7.3 (5.1)	<0.001
pH ≤ 4 (%) upright	66	12.0 (11.3)	60	8.5 (6.1)	0.013
pH ≤ 41 (%) supine	66	6.8 (11.0)	59	5.4 (6.9)	0.48
Total episodes	66	170.8 (181.6)	64	100.4 (105.9)	<0.001
Longest episode (min)	66	23.9 (22.1)	63	21.1 (20.0)	0.28

* P value versus baseline Wilcoxon signed-rank test

**Table 7:** Summary of esophageal manometry data

Physiologic measurement	Baseline	Month 6	*P* value*
LES pressure (mmHg)			
*N*	64	58	
Mean (SD)	11.6 (8.6)	12.5 (8.0)	
Median	9.85	11.15	0.43
LES length (cm)			
*N*	62	58	
Mean (SD)	40.9 (17.7)	36.5 (18.7)	
Median	40.0	35.0	0.16
Peristaltic amplitude (mmHg)			
*N*	64	58	
Mean (SD)	78.8 (35.8)	80.7 (40.1)	
Median	70.0	68.5	

* *P* value versus baseline Wilcoxon signed-rank test

**Table 8:** Hill grades at baseline: pre- and post-procedure

	6-month post-procedure		
	Grade ≤2	Grade >2	Total
Baseline (pre-procedure)			
Grade ≤2	21	1	22
Grade >2	40	3	43
Total	61	4	65

### Safety results and side effects

At 6 months there were no residual serious adverse events related to the device or the procedure. In the final 48/72 subjects enrolled, there were two SAEs, neither of which required intervention. One was rated mild in severity requiring additional inpatient observation for elevated C-Reactive Protein (CRP). Another was non-procedure related involving an overnight hospitalization for a psychiatric emergency 35 days post-procedure.

However, there were eight SAEs recorded in the first 24 subjects (Table 9). Four were rated as mild and transitory. Three of these were kept in hospital for observation an additional 24 h over the defined limit of 72 h, and one was readmitted for 1 day. All four had normal endoscopies and radiological studies. Two SAEs were rated as moderate, with findings of pneumomediastinum and/or pneumoperitoneum. Both patients were asymptomatic and recovered uneventfully without intervention, but were hospitalized for observation an additional 4 and 14 days, respectively. Two SAEs were rated as severe and required intervention. The first involved a subject who returned to the hospital 3 days post-procedure with empyema and pneumothorax, although the immediate post-procedure chest roentgenogram was normal. A perforation was not demonstrated on endoscopy or by radiological (contrast CT and fluoroscopy) studies, but the drained fluid had high amylase concentration indicative of an esophageal leak. This patient underwent chest tube and antibiotic therapy and recovered after a hospitalization of 22 days. There were no long-term sequelae in follow-up. The subject had severe retching post-anesthesia, which likely caused excessive tension on one or both of the stapling sites. The second severe SAE was an upper gastrointestinal hemorrhage which presented 8 days after the procedure. The patient was re-hospitalized for 72 h and received a two-unit transfusion. Endoscopy did not reveal a source, and recovery was complete.

**Table 9:** Summary of SAEs

SAE	Sex	Age (years)	Days after procedure	Duration (days)	Rating	Life threatening	SAE description
*N* = 24 subjects enrolled							
1	M	51.1	2	2	Mild	No	Pain and fever
2	M	55.1	1	4	Mild	No	Pain and fever
3	M	29.1	1	14	Moderate	No	pneumomediastinum
4	F	66.9	16	1	Mild	No	Pain in the thorax
5	F	55.3	1	4	Moderate	No	Pneumothorax pneumoperitoneum
6	M	37.2	8	1	Mild	No	Viral infection
7	M	38.1	3	22	Severe	No	Pneumothorax Pleural effusion Esophageal leak
8	M	32.1	8	3	Severe	No	Upper GI bleed
*Interim safety analysis*: *implementation of protocol and device changes*							
*N* = 48 subjects enrolled							
9	M	45.8	35	1	Severe	No	Suicidal behavior
10	F	61.3	4	1	Mild	No	Fever, elevated CRP

The interim review of these early SAEs resulted in protocol and device changes implemented after the first 24 subjects to mitigate risks. It was noted that six of the SAEs were in subjects that received only two staplings, therefore an additional stapling was encouraged with the aim of reducing stress at an individual stapling site. The protocol was also amended to require prophylactic therapy to prevent immediate post-operative retching which can also stress stapling sites, and to require a chest X-ray to confirm no leaks are present prior to discharge. In addition, device changes were made to prevent air insufflation during screw insertion in order to prevent the tendency of air to leak into the peritoneum around the screws before the staples are formed. Following these amendments and procedural changes there were no further cases of leak or pneumomediastinum in the next 48 subjects enrolled.

The most common adverse events reported were chest pain in 22 % (16/72) and sore throat in 21 % (15/72) of patients. AE’s occurring in more than 5 % of patients was atelectasis, pain in the shoulder, and increased belching. All resolved spontaneously and the majority were reported in the immediate post-operative period (Table 10). There were no reports of dysphagia, bloating, or inability to belch.

**Table 10:** Device- and/or procedure-related AEs in 5 % or more of patients

Adverse event	Post-procedure to discharge	1 week (6–10 days post-procedure)	2 weeks (±4 days)	Total	Events/subject (*N* = 72)
Chest pain	15	1		16	22 %
Sore throat	15			15	21 %
Atelectasis	6			6	8.3 %
Shoulder pain	5		2^a^	5	6.9 %
Increase belching	4			4	5.5 %

^a^1 event occured 19 days following the procedure

## Discussion

PPI therapy and LNF have been the mainstay of GERD treatment. Their limitations and adverse effects have led to the development of currently marketed alternative therapies including Stretta™, Esophyx™, Linx™ and others [26–30].

Unlike other procedures, the endostapler closely mimics surgical anterior fundoplication through transoral stapling. The device incorporates a video camera for direct visualization during insertion and staple site selection and ultrasound to determine when a proper stapling gap is achieved. In this report, the majority of patients treated with the endostapler had improved symptom control of GERD and no longer needed daily PPI therapy at 6 months.

Additional exploratory analysis revealed that the GERD-HRQL sub score of heartburn-related questions was also improved for post-procedure patients even as compared with those on PPI pre-procedure. These data further serves to illustrate that the procedure can relieve heartburn symptoms and provide an effective alternative to chronic PPI use.

The procedure may not preclude future surgical fundoplication; two subjects elected to have a LNF after conclusion of the study and this was accomplished without difficulty. The current protocol mandated hospitalization and observation of all subjects; however, the procedure may eventually be performed in an outpatient setting as experience increases.

Important limitations in the design of this study include a short follow-up period and the lack of a sham or control group. Further study is planned. Three-year follow-up data is being collected and will be reported. The study excluded the subset of patients with relatively common GERD complications such as large hiatal hernia, severe erosive esophagitis, and symptoms non-responsive to PPI therapy, Barrett’s esophagus and esophageal motility disorders. New studies will be necessary to determine the safety and efficacy of the device in such patients.

There were no post-procedure reports of common problems seen with LNF such as gas bloating, inability to belch or vomit, and dysphagia. Most adverse events were reported in the immediate peri-operative period and were most commonly chest pain or sore throat.

There were no run-in procedures for this study. As such, serious adverse events were concentrated in the first 24 subjects. The use of anti-retching prophylaxis, increased number of staplings, and insufflation control during the screw deployment resulted in a much improved safety profile in the remaining 48 subjects enrolled with no additional cases of leakage or pneumomediastinum reported. Trials using CO2 insufflation would be of interest. As with many new procedures, the safety and effectiveness profile should continue to improve as experience is gained and a newer generation device is introduced.

In conclusion, this study reports early experience of an endoscopic stapler for patients with chronic GERD. As a result of this study, the system was FDA cleared and CE marked for endoscopic placement of surgical staples in the soft tissue of the esophagus and stomach in order to create an anterior partial fundoplication for treatment of symptomatic chronic GERD in patients who require and respond to pharmacological therapy. The procedure is an option to offer patients looking for reduction or discontinuance of GERD medical therapy, and avoidance of problematic side effects associated with incisional therapies such as LNF. Longer term follow-up of this patient group is underway. An improved version of the system has recently been cleared with an improved user interface. Additional studies in a larger patient population are needed and will assess this new system for safety and effectiveness before conclusions of the procedure durability can be established.
